# Reliability and normative values for the foot mobility magnitude: a composite measure of vertical and medial-lateral mobility of the midfoot

**DOI:** 10.1186/1757-1146-2-6

**Published:** 2009-03-06

**Authors:** Thomas G McPoil, Bill Vicenzino, Mark W Cornwall, Natalie Collins, Meghan Warren

**Affiliations:** 1 Gait Research Laboratory, Program in Physical Therapy, Northern Arizona University, Flagstaff, Arizona, USA; 2 Department of Physiotherapy, University of Queensland, St. Lucia, Brisbane, QLD 4072, Australia

## Abstract

**Background:**

A study was conducted to determine the reliability and minimal detectable change for a new composite measure of the vertical and medial-lateral mobility of the midfoot called the foot mobility magnitude.

**Methods:**

Three hundred and forty-five healthy participants volunteered to take part in the study. The change in dorsal arch height between weight bearing and non-weight bearing as well as the change in midfoot width between weight bearing and non-weight bearing were measured at 50% of total foot length and used to calculate the foot mobility magnitude. The reliability and minimal detectable change for the measurements were then determined based on the assessment of the measurements by three raters with different levels of clinical experience.

**Results:**

The change in dorsal arch height between weight bearing and non-weight bearing, midfoot width between weight bearing and non-weight bearing, and the foot mobility magnitude were shown to have high levels of intra-rater and inter-rater reliability. Normative data are provided for the left and right feet of both the female (n = 211) and male (n = 134) subjects.

**Conclusion:**

While the measurements of navicular drop and drift have been used as a clinical method to assess both the vertical and medial-lateral mobility of the midfoot, poor to fair levels of inter-rater reliability have been reported. The results of the current study suggest that the foot mobility magnitude provides the clinician and researcher with a highly reliable measure of vertical and medial-lateral midfoot mobility.

## Background

Foot posture and mobility are critical components in the evaluation of foot function, in the prescription of foot orthoses, as well as in planning for the management of various foot pathologies. The clinical assessment of foot posture and mobility require the use of simple and minimally invasive techniques that can be easily and efficiently performed as well as demonstrate high levels of within and between rater reliability. The measurement of foot posture allows the clinician to classify an individual's foot shape, most commonly by describing the height of the medial longitudinal arch (i.e.; high, normal, or low). Numerous techniques for the assessment of foot posture have been described in the literature including the arch index [1], the bony arch index [2], valgus index [3], longitudinal arch angle [4,5], and the arch height index [6]. While the authors of these studies have described good to high levels of intra-rater and inter-rater reliability for these various foot posture assessment techniques, the cohorts used to establish normative values for these different methods have been less than 200 subjects. More recently, McPoil et al did publish normal values for the arch height index based on 850 healthy subjects [7]. They reported not only high levels of intra-rater and inter-rater reliability, but also validated the measurement using radiographs. Although these various measures of foot posture allow the clinician to categorize an individual's static foot posture, the major limitation for any technique that is used to classify arch height is that such a categorization does not take into account the mobility of the foot. As noted by Menz [8], "the quantity and quality of motion in the joints of the foot cannot be determined simply by observing arch height."

Two assessment techniques commonly described in the literature to assess foot mobility are navicular drop and navicular drift. Navicular drop, first described by Brody, is a measure of the sagittal plane mobility of the midfoot by quantifying the vertical change in the height of the navicular tuberosity [9]. The most studied of the two techniques, previous investigations have reported that navicular drop has high levels of intra-rater reliability, poor to moderate levels of inter-rater reliability and a lack of normative data from a large cohort of healthy individuals [10-13]. The most prominent issues explaining the lower levels of inter-rater reliability for navicular drop are the identification of the navicular tuberosity bony landmark as well as the consistency of placing the subtalar joint in neutral position using palpation. In response to the problems associated with the navicular drop measurement, McPoil et al described an alternative measurement that assesses the change in dorsal arch height from weight bearing to non-weight bearing [14]. They reported that the change in dorsal arch height from weight bearing to non-weight bearing had good to high levels of intra-rater and inter-rater reliability, as well as validity using radiographs as the criterion measure. While the change in dorsal arch height from weight bearing to non-weight bearing would appear to offer the clinician a reliable and valid alternative to the navicular drop test, the authors note that the measurement method may be too time consuming for clinicians since the capture and modification of digital images of the foot are required.

Menz was one of the first to suggest that in addition to sagittal plane movement, medial-lateral movement of the midfoot should also be assessed by using the navicular drift measurement [8]. Menz noted that although the largest amount of motion occurred in the sagittal plane, it would seem reasonable to suggest that measurement of medial drift, or transverse plane movement, of the navicular may provide further insight in the mechanics of the talonavicular joint. The assessment of both sagittal and medial-lateral movement of the midfoot would also appear warranted based on dynamic motion studies assessing navicular movement during walking. Cornwall and McPoil assessed three-dimensional movement of the navicular bone in 106 healthy subjects using an electromagnetic tracking system [15]. They reported that in relation to resting standing posture, the maximum total excursion of the navicular bone calculated as the resultant of both medial-lateral and sagittal (vertical) movement was 0.8 ± 0.3 cm. Thus, it would appear that in order to fully understand the movement of the midfoot, as measured using the navicular bone as the reference point, that medial-lateral as well as sagittal plane movements should be assessed.

The results of previous studies attempting to evaluate the consistency of the navicular drift measurement have been mixed. Vinicombe et al had five podiatrists with three to seven years of experience assess the navicular drift of 20 healthy subjects on two different occasions [16]. Each podiatrist received three 1-hour training sessions to ensure consistency of the measurement technique used to assess navicular drift. Intraclass correlation coefficients ranged from 0.31 to 0.62, with intra-rater reliability slightly better than inter-rater reliability. The mean for navicular drift was 0.7 ± 0.3 cm with the standard error of the measurement ranging from ± 0.3 to ± 0.5 cm. Vinicombe et al concluded that the method they utilized to measure navicular drift was only moderately reliable and that clinicians should be cautious in light of the error associated with the measurement [16].

Billis et al also assessed the reliability of navicular drift on the left foot of 26 healthy subjects using two physiotherapists [17]. They reported that the test-retest reliability for navicular drift, based on data collected for a different study, was 0.95 to 0.99. The authors, however, note in their paper that these values represent good intra-rater reliability. Thus, it is unclear if the authors actually did attempt to assess the inter-rater reliability for the navicular drift measurement. The mean for navicular drift reported by Billis et al was 1.01 ± 0.3 cm [17]. Brushoj et al proposed the use of a foot line test, rather than assessing navicular drift, to assess the medial-lateral movement of the midfoot by using the most medial prominence of the navicular tuberosity compared to the position of the forefoot and rearfoot [18]. Two raters, a physiotherapy student and a physician, assessed the foot line test on 130 healthy males after practicing the technique for 30 minutes. While the authors reported high levels of intra-rater (0.94 to 0.95) and good levels of inter-rater (0.83 to 0.86) reliability, the standard deviations and the standard error of the measurements (SEM) were quite large. The mean foot line test for rater 1 was 0.33 ± 0.34 cm with an SEM of 0.14 cm. The mean foot line test for rater 2 was 0.39 ± 0.32 cm with an SEM of 0.13 cm. In addition, the authors reported that the mean difference between the two raters was significant [18]. One possible explanation for these results is that foot line test requires the rater to physically mark the location of the navicular tuberosity as well as the location of the first metatarsophalangeal joint. As previously noted, one of the issues associated with the lack of consistency in measuring navicular height is the problem of raters having difficulty agreeing on the location of bony landmarks using palpation.

While it is obvious that the clinical assessment of foot mobility requires a measure of both sagittal (vertical) and medial-lateral movement of the midfoot, based on the poor to moderate inter-rater reliability values previously reported for both navicular drop and drift there is a need for an alternative method to assess these two components of foot mobility. McPoil et al have demonstrated that the change in dorsal arch height from weight bearing to non-weight bearing was a reliable and valid measure to replace the assessment of navicular drop, but the development of a less time-intensive method to perform this assessment in the clinic is required [14]. If this could be accomplished, the difference in dorsal arch height between weight bearing and non-weight bearing could provide an acceptable method to assess sagittal or vertical mobility of the midfoot. McPoil et al have also previously described the use of the change in midfoot width from weight bearing to non-weight bearing as a measure of medial-lateral mobility of the midfoot [19]. Since this was a pilot study, however, only minimal information on the reliability of the measurement was provided. In a recently published paper attempting to determine predictors that could be used to identify patients with patellofemoral pain who would benefit from the use of foot orthoses, Vicenzino et al identified the change in midfoot width from weight bearing to non-weight bearing as one of four predictors[20]. While the change in midfoot width between weight bearing and non-weight bearing as described by McPoil et al could provide an acceptable method for assessing the medial-lateral mobility of the midfoot, further assessment of the reliability of the measurement is also required. If the change in dorsal arch height between weight bearing and non-weight bearing as well as change in midfoot width between weight bearing and non-weight bearing were found to have acceptable levels of intra-rater and inter-rater reliability, these two measures could be used to calculate a foot mobility magnitude measure which would provide the clinician and researcher with a composite value of vertical and medial-lateral mobility of the midfoot. Thus, the purposes of this study were to: 1) describe the techniques used to measure the change in dorsal arch height between weight bearing and non-weight bearing (DiffAH) as well as the change in midfoot width between weight bearing and non-weight bearing (DiffMFW); 2) determine the reliability of the measurements required to calculate the DiffAH and DiffMFW; and 3) report normative values for DiffAH, DiffMFW, and the Foot Mobility Magnitude (FMM) measurements.

## Methods

### Participant characteristics

Three hundred and forty-five participants (211 females and 134 males) volunteered to participate in the study. Participants were recruited from the Northern Arizona University population and the surrounding Flagstaff, Arizona community. All participants met the following inclusion criteria: 1) no history of congenital deformity in the lower extremity or foot; 2) no previous history of lower extremity or foot fractures; 3) no systemic diseases that could affect lower extremity or foot posture; and 4) no history of trauma or pain to either foot, lower extremity, or lumbosacral region at least 12 months prior to the start of the investigation. The mean age of the 345 participants was 26.3 ± 5.6 years with a range of 18 to 61 years. The mean age of the female and male participants was 26.2 ± 5.7 and 27.3 ± 5.5 years, respectively. Although no standardized "warm-up" protocol was used for the participants prior to data collection, each participant had been weight bearing and ambulating for at least 1 hour while conducting their normal activities of daily living prior to participating in the study.

### Instrumentation

**Figure 1:**
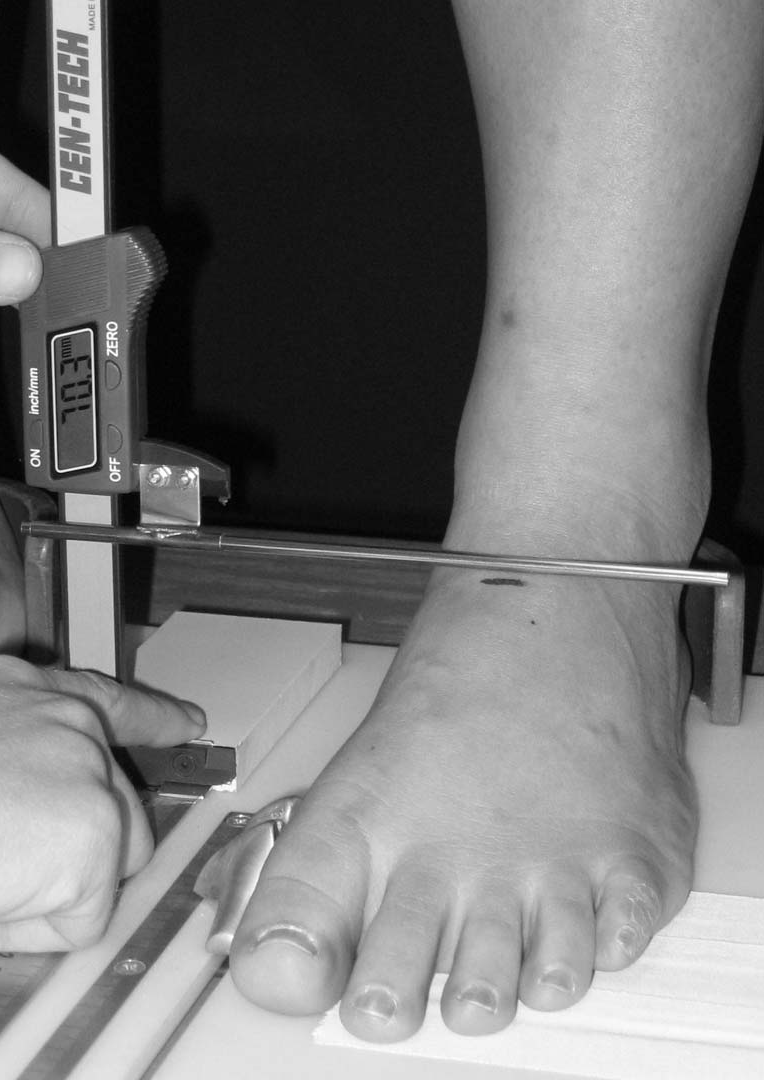
**Digital Gauge used to measure dorsal arch height in weight bearing**.

**Figure 2:**
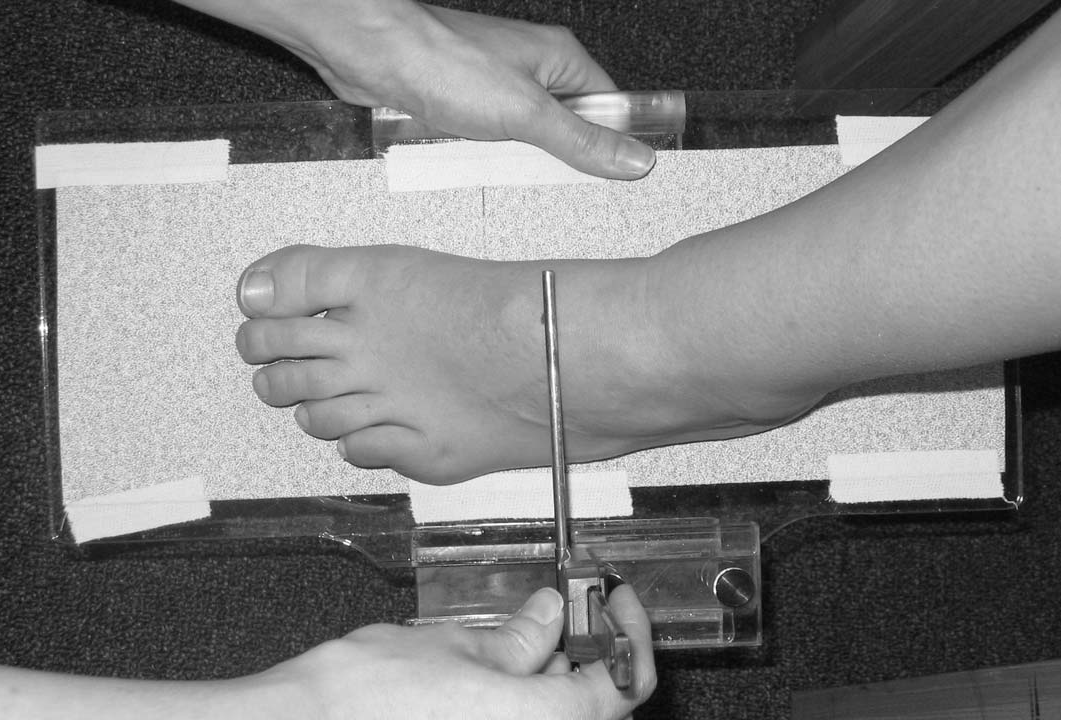
**Plastic portable platform with digital gauge used to measure non-weight bearing dorsal arch height**.

**Figure 3:**
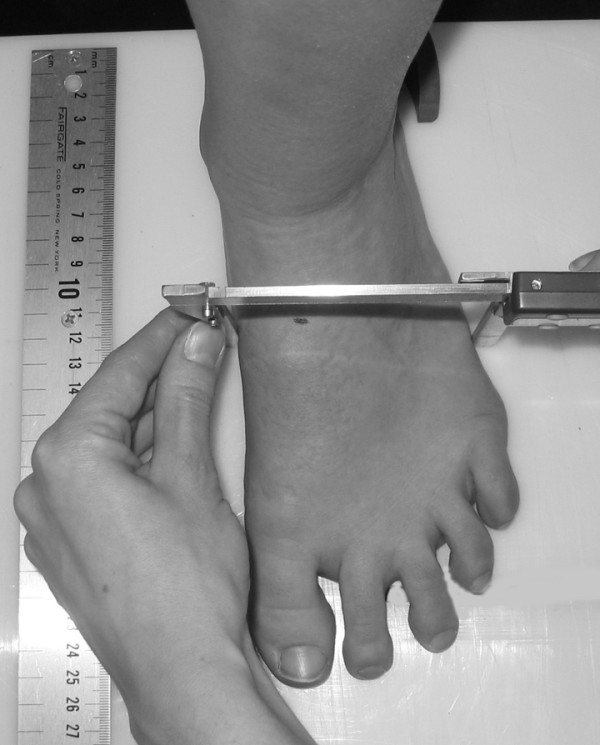
**Use of modified digital caliper to measure midfoot width in weight bearing**.

Three instruments were manufactured for the study to permit the measurement of both arch height and midfoot width. The weight bearing arch height gauge consisted of a digital caliper (Model #93293, Cen-Tech, Harbor Freight Tools, Carmarillo, CA 93011) with the fixed point attached to a 1.2 × 5.0 × 10.0 cm plastic block to hold the caliper in a vertical position and a sliding metal rod attached to the moving point of the caliper to permit the assessment of arch height (see Figure 1). To assess non-weight bearing arch height, a second identical digital caliper with the same modifications as described for the weight bearing arch height gauge was mounted to a 0.5 × 12.0 × 41.0 cm plastic portable platform (see Figure 2). The plastic block attached to the fixed point of the caliper was attached to the portable platform so that it could be moved in order to permit proper alignment of the sliding metal rod to different foot lengths. To enhance the participant's awareness of the platform touching the plantar surface of their foot, 80-grit sandpaper was taped to the superior surface of the portable platform. A third digital caliper (Model # S54-101-150-2, Fowler Equipment, Newton, MA 02466) was modified, to permit the measurement of midfoot width in both weight bearing and non-weight bearing by attaching 0.03 × 0.8 × 9.0 cm metal plates to both the fixed and the moving points of the caliper (see Figure 3).

### Procedures

**Figure 4:**
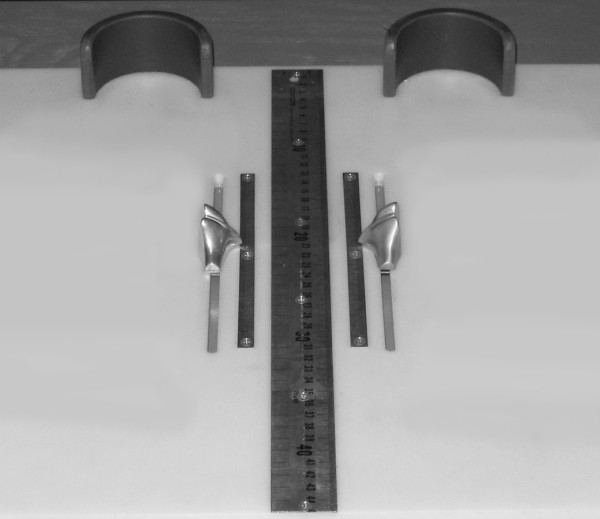
**Foot measurement platform**.

**Figure 5:**
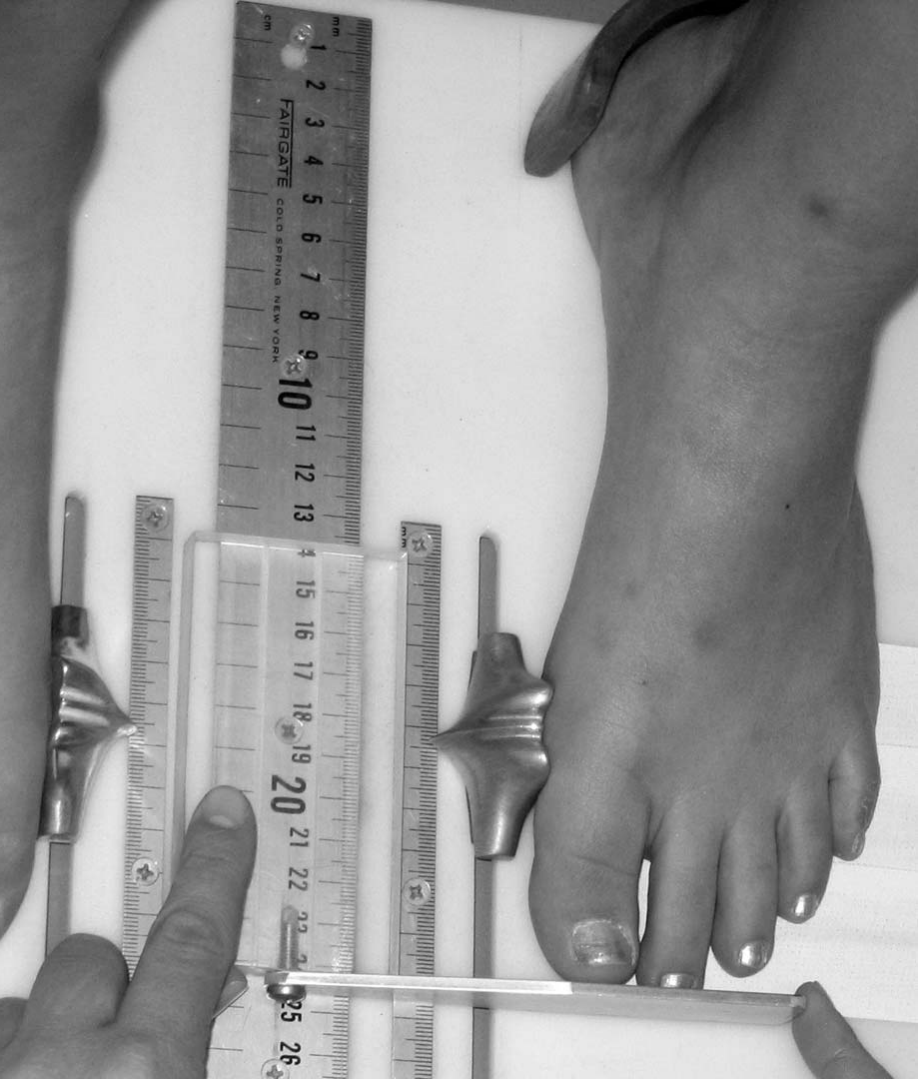
**Placement of the forefoot using first metatarsophalangeal joint indicator and measurement of total foot length**.

Each subject was asked to stand on a previously described foot measurement platform so that total foot length, dorsal arch height, and midfoot width could be measured in bilateral lower limb weight bearing (see Figure 4) [7]. Prior to obtaining the standing measurements, each subject was positioned on the foot measurement platform with both heels placed in left and right heel cups that were positioned 15.24 cm apart. Next, the sliding first metatarsophalangeal joint indicator was positioned over the medial prominence of the first metatarsal head to ensure consistent forefoot placement on the platform (see Figure 5). Once the subject was properly positioned on the platform, the subject was instructed to place equal weight on both feet so that the weight bearing measurements could be obtained. Total foot length was first measured by placing the sliding bar on the centered metal ruler attached to the platform and moving the bar to just touch the longest toe, usually the hallux, of each foot (see Figure 5). Next, the dorsal arch height at 50% of total foot length was measured bilaterally using the weight bearing arch height gauge previously described. To determine the point of 50% of total foot length, the previously measured total foot length was divided in half and the dorsum of both feet were marked at the 50% length point using a water-soluble pen. The sliding metal rod of the weight bearing height gauge was then positioned over the 50% length mark and the vertical height from the top of the platform to the dorsum of the foot was measured (see Figure 1). Next, the caliper designed to assess midfoot width was positioned so that the edges of the two metal plates attached to each point of the caliper where aligned laterally and medially to the 50% length point on the dorsum of the foot (see Figure 3). The rods where then moved together until rods on both the lateral and the medial side of 50% length point just made contact with the skin. Once both rods made contact with the skin, the distance was recorded. The use of 50% of the total foot length for both measurements was based on the results of previous research assessing the consistency of the arch height index [6,7].

**Figure 6:**
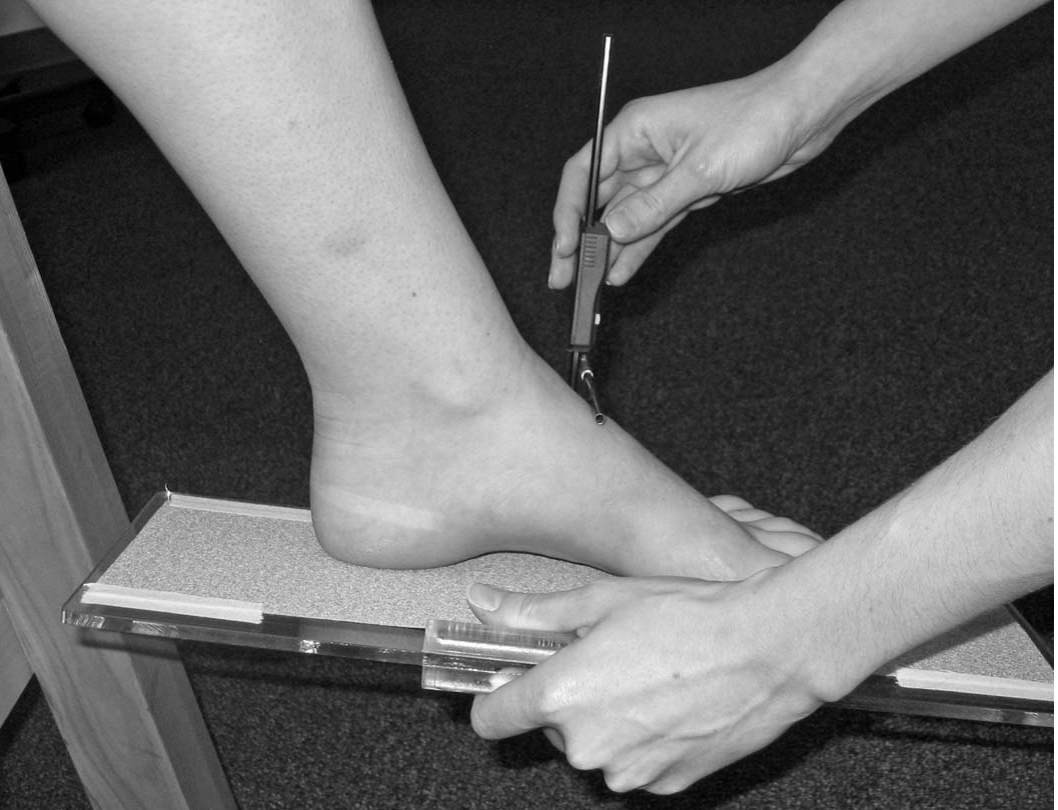
**Placement of the portable platform under foot plantar surface to permit measurement of non-weight bearing dorsal arch height**.

**Figure 7:**
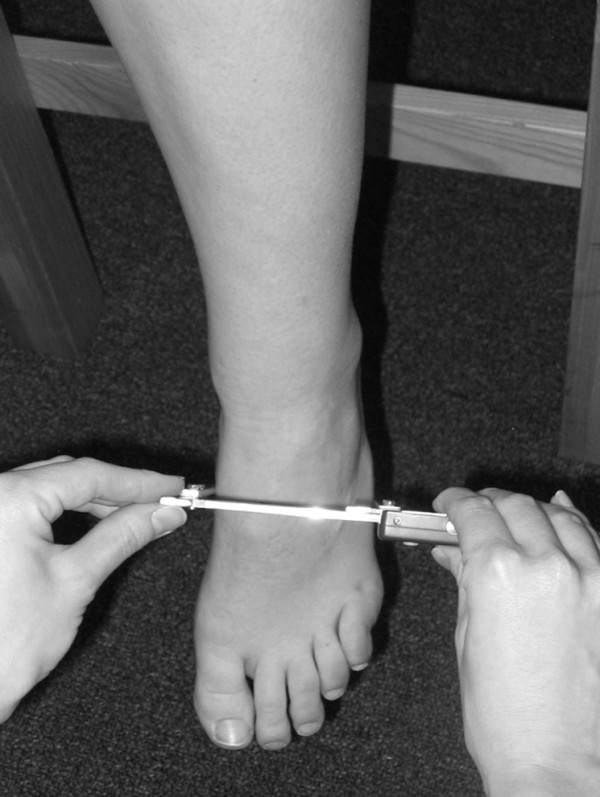
**Measurement of non-weight bearing midfoot width using digital width caliper**.

Following the completing of the weight bearing measurements, each subject was asked to sit on the end of a table so that both lower legs were hanging in a perpendicular position to the floor with the feet non-weight bearing and the ankles slightly plantar-flexed. In this position, the non-weight bearing measurements of dorsal arch height and midfoot width were recorded. To assess dorsal arch height in non-weight bearing, the portable platform was positioned under, but without touching the plantar surface of the right foot for each subject. As the portable platform was then moved upward to make contact with the plantar surface of the foot, the subject was instructed to state when they sensed the portable platform "just touching" the plantar surface of the heel, lateral forefoot and medial forefoot of the right foot simultaneously. The subject was told to indicate to the rater if they felt that the portable platform was forcibly pushing their foot into ankle dorsiflexion. If this did happen, the procedure was stopped and repeated so that the subject only sensed that the portable platform was just touching the plantar surface of the right foot. When the subject indicated that the portable platform was "just touching" the plantar surface of their right foot, the vertical digital caliper attached to the portable platform was positioned so that the sliding metal rod could be placed over the 50% foot length mark on the dorsum of the foot (see Figure 6). Once the sliding metal rod of the vertical digital caliper was positioned over the 50% foot length mark, the vertical height from surface of the portable platform to the dorsum of the foot was measured (see Figure 2). To measure the midfoot width in non-weight bearing, the width caliper was positioned so that the edges of the two metal plates attached to each pin of the caliper where aligned laterally and medially to the 50% length point on the dorsum of the right foot. The metal plates were then moved together until rods on both the lateral and the medial side of 50% length point made contact with the skin of the right foot (see Figure 7). Once both rods made contact with the skin, the midfoot width distance was recorded. The non-weight bearing measures were then repeated for the left foot.

A preliminary pilot study conducted by the authors indicated that acceptable reliability coefficients (ICC > 0.90) required only a single measure in weight bearing but the average of two measures in non-weight bearing. Thus in the current study, all weight bearing measurements were only recorded once and all non-weight bearing measurements were recorded twice. All measurements were obtained on the 345 participants by the same rater (rater 1) and were recorded in centimeters. The required time to complete all the measures for each subject ranged from 6 to 8 minutes. To determine DiffAH, the dorsal arch height in weight bearing was subtracted from the average of the two measures of dorsal arch height in non-weight bearing. To determine the DiffMFW, the average of the two midfoot width measures recorded in non-weight bearing were subtracted from the single weight bearing midfoot width. The method used to calculate the foot mobility magnitude (FMM), was based on the Pythagorean theorem. The DiffAH represented the vertical side and the DiffMFW represented the horizontal side of a right triangle. To calculate the composite FMM (the hypotenuse of the right triangle), the following formula was used:

FMM = √(DiffAH)^2 ^+ (DiffMFW)^2^

### Determination of reliability

To establish intra-rater and inter-rater reliability for the measurements, three raters were asked to assess the left and right feet of 12 randomly selected participants. The raters performing the measurements were three physical therapists with a minimum of 2 years clinical experience (mean experience 16 years; range 2 to 30 years). Each rater attended a single one-hour training session to receive verbal instructions as well as to practice the techniques to ensure that they were taking the measurements correctly. The reliability data collection consisted of two sessions, one-week apart, in which each rater performed all five measurements on all 12 subjects. At each session, each rater performed all five measurements on both feet of each subject twice with at least 10 minutes separating the two sets of measurements. In addition to inter-rater reliability, both within-session and between-session reliability could also be determined for each rater. Each rater was blinded from all measurements and the mark placed over the dorsum of each foot was removed after each set of measurements to prevent subsequent rater bias. The left and right feet for all 12 subjects were treated as independent observations so that the analysis of reliability was conducted on 24 feet.

### Statistical analysis

Intraclass correlation coefficients (ICC) were calculated to determine the consistency of each rater to repeatedly perform the measurements individually (intra-rater; ICC_3,1_) for both within-session and between-session as well as in comparison to the other raters (inter-rater; ICC_2,3_) [21]. For the between-session reliability assessment, the values for the five measurements collected twice in each session were averaged. For the inter-rater reliability assessment, in addition to the five measurements made by each rater, ICC values were calculated for DiffAH, DiffMFW, and FMM. The level of reliability for the ICC was classified using the characterizations reported by Landis and Koch [22]. These characterizations were:*slight*, if the correlation ranged from 0.00 to 0.20; *fair*, if the correlation ranged from 0.21 to 0.40; *moderate*, if the correlation ranged from 0.41 to 0.60; *substantial*, if the correlation ranged from 0.61 to 0.80; and *almost perfect*, if the correlation ranged from 0.81 to 1.00.

Although the ICC is a well accepted measure of reliability, it is difficult to interpret ICC values since they are dependent on the variability of the group being assessed and thus, may not transfer to different patient populations [23]. Thus in addition to ICC values, the standard error of the measurement (SEM) was also calculated as another index of reliability. The SEM is a number in the same units as the original measurement that represents the way a single score would vary if the five measurements used in this study were measured more than once [24]. In addition the SEM was used to calculate the minimal detectable change (MDC) for all measurements. The MDC reflects the amount of change required for change to be considered "real," over and above measurement error. In theory, change scores greater than the MDC would have less than a 5% chance of being a change due to chance or measurement errors alone, if 95% confidence limits (MDC95% = 1.96v2 × SEM or 2.77 × SEM) are used [25]. Thus, change scores greater than the MDC can be considered with reasonable confidence to represent true change and as such, represent the lower boundary of a potentially meaningful change (i.e., the minimal clinically important difference) [26].

In addition to descriptive statistics, the Shapiro-Wilk W test was used as the formal test of normality for all measurements on the female and male feet. *T *tests were performed to determine whether differences existed between the females or males and the left or right feet for all measurements. Because of the multiple comparisons conducted using t tests, an alpha level of .01 was established for all tests of significance to avoid a possible type I error.

## Results

The within-session and between-session ICC and SEM values for all three raters are shown in Tables 1 and 2. The within-session and between-session ICC values for all three raters ranged from 0.97 to 0.99 for foot length, midfoot width in weight bearing, dorsal arch height in weight bearing, midfoot width in non-weight bearing, and dorsal arch height in non-weight bearing, with the SEM values ranging from 0.03 to 0.09 cm. The ICC, SEM and MDC95% values for inter-rater reliability amongst all three raters for total foot length, midfoot width in weight bearing, dorsal arch height in weight bearing, midfoot width in non-weight bearing, dorsal arch height in non-weight bearing, DiffAH, DiffMFW, and FMM are listed in Table 3. The inter-rater ICC values ranged from 0.83 to 0.99 for all eight measurements with SEM values ranging from 0.04 to 0.13 cm. The inter-rater MDC95% values ranged from a high of 0.37 to low of 0.10 cm. Since the same rater (rater 1 from the reliability portion of the study) performed all the measurements on the 345 participants that were used to establish normative data, the ICC and SEM values for the left and right feet of the 12 subjects from the reliability portion of the study are provided for this rater in Table 4. The normative data MDC95% values listed in Tables 5 and 6 were calculated using the SEM values determined from the reliability portion of the study for rater 1.

**Table 1:** Within-session intra-rater reliability coefficients (ICC) and standard error of the measurement (SEM)

	**Rater 1** **(30 years experience)**			**Rater 2** **(16 years experience)**			**Rater 3** **(2 years experience)**		
	**ICC**	**Mean (cm)**	**SEM (cm)**	**ICC**	**Mean (cm)**	**SEM (cm)**	**ICC**	**Mean (cm)**	**SEM (cm)**

**Foot Length**	0.99	25.96	0.04	0.99	26.05	0.04	0.99	25.96	0.04
**Midfoot Width WB**	0.98	8.82	0.04	0.99	8.88	0.03	0.99	8.61	0.04
**Dorsal Arch Hgt WB**	0.98	6.52	0.03	0.98	6.53	0.03	0.98	6.52	0.03
**Midfoot Width NWB #1**	0.98	7.71	0.05	0.97	7.78	0.07	0.97	7.84	0.06
**MIdfoot Width NWB #2**	0.98	7.70	0.05	0.97	7.75	0.07	0.97	7.77	0.06
**Dorsal Arch Hgt NWB #1**	0.98	7.97	0.04	0.97	7.84	0.05	0.98	7.91	0.03
**Dorsal Arch Hgt NWB #2**	0.99	7.93	0.03	0.98	7.78	0.05	0.98	7.87	0.04

**Note**: WB = Weight Bearing; NWB = Non-Weight Bearing

**Table 2:** Between-session intra-rater reliability coefficients (ICC) and standard error of the measurement (SEM)

	**Rater 1** **(30 years experience)**			**Rater 2** **(16 years experience)**			**Rater 3** **(2 years experience)**		
	**ICC**	**Mean (cm)**	**SEM (cm)**	**ICC**	**Mean (cm)**	**SEM (cm)**	**ICC**	**Mean (cm)**	**SEM (cm)**

**Foot Length**	0.99	25.97	0.06	0.99	26.08	0.06	0.99	25.98	0.05
**Midfoot Width WB**	0.99	8.83	0.05	0.99	8.91	0.05	0.98	8.65	0.06
**Dorsal Arch Hgt WB**	0.98	6.54	0.03	0.98	6.54	0.05	0.98	6.54	0.03
**Midfoot Width NWB #1**	0.98	7.72	0.05	0.98	7.75	0.09	0.97	7.82	0.06
**MIdfoot Width NWB #2**	0.97	7.71	0.06	0.97	7.73	0.08	0.98	7.78	0.05
**Dorsal Arch Hgt NWB #1**	0.98	7.96	0.05	0.98	7.86	0.08	0.98	7.90	0.05
**Dorsal Arch Hgt NWB #2**	0.99	7.92	0.05	0.98	7.73	0.09	0.98	7.86	0.06

**Note**: WB = Weight Bearing; NWB = Non-Weight Bearing

**Table 3:** Inter-rater reliability coefficients (ICC), standard error of the measurement (SEM), and minimal detectable change (MDC) scores

	**Rater 1** **(Mean ± SD)** **(cm)**	**Rater 2** **(Mean ± SD)** **(cm)**	**Rater 3** **(Mean ± SD)** **(cm)**	**ICC**	**ICC** **95% CI**	**SEM** **(cm)**	**MDC95%** **(cm)**
**Foot Length**	25.97 ± 1.86	26.08 ± 1.88	25.98 ± 1.90	0.99	0.98 – 1.00	0.06	0.15
**Midfoot Width WB**	8.83 ± 0.93	8.91 ± 0.93	8.65 ± 0.93	0.99	0.98 – 1.00	0.05	0.14
**Dorsal Arch Hgt WB**	6.54 ± 0.53	6.54 ± 0.51	6.54 ± 0.54	0.99	0.98 – 1.00	0.04	0.10
**Midfoot Width NWB**	7.71 ± 0.68	7.72 ± 0.73	7.80 ± 0.77	0.97	0.95 – 0.98	0.11	0.31
**Dorsal Arch Hgt NWB**	7.94 ± 0.68	7.78 ± 0.66	7.88 ± 0.64	0.99	0.98 – 1.00	0.06	0.17
**Diff Midfoot Width**	1.13 ± 0.31	1.19 ± 0.32	0.85 ± 0.26	0.83	0.66 – 0.93	0.13	0.37
**Diff Dorsal Arch Hgt**	1.40 ± 0.21	1.25 ± 0.24	1.33 ± 0.19	0.89	0.78 – 0.96	0.07	0.20
**Foot Mobility Magnitude**	1.81 ± 0.31	1.73 ± 0.34	1.59 ± 0.22	0.86	0.73 – 0.95	0.11	0.31

**Note**: WB = Weight Bearing; NWB = Non-Weight Bearing; 95%CI = 95% confidence intervals

**Table 4:** Intra-rater reliability (ICC), standard error of the measurement (SEM), and minimal detectable change (MDC) scores for RATER 1

	**Day 1** **(Mean ± SD)** **(cm)**	**Day 2** **(Mean ± SD)** **(cm)**	**ICC**	**ICC** **95% CI**	**SEM** **(cm)**	**MDC95%** **(cm)**
**LEFT FOOT**						
						
Foot Length	25.96 ± 1.85	26.02 ± 1.86	0.99	0.99 – 1.00	0.11	0.32
Midfoot Width WB	8.82 ± 0.92	8.83 ± 0.94	0.99	0.98 – 1.00	0.09	0.24
Dorsal Arch Hgt WB	6.53 ± 0.55	6.55 ± 0.50	0.98	0.95 – 0.99	0.07	0.19
Midfoot Width NWB	7.72 ± 0.68	7.70 ± 0.68	0.98	0.95 – 0.98	0.10	0.27
Dorsal Arch Hgt NWB	7.94 ± 0.66	7.93 + 0.66	0.98	0.96 – 1.00	0.08	0.23
Diff Midfoot Width	1.11 ± 0.32	1.14 ± 0.29	0.87	0.70 – 0.96	0.11	0.30
Diff Dorsal Arch Hgt	1.41 ± 0.20	1.39 ± 0.22	0.91	0.79 – 0.97	0.06	0.17
Foot Mobility Magnitude	1.81 ± 0.30	1.80 ± 0.32	0.93	0.84 – 0.98	0.07	0.18
						
**RIGHT FOOT**						
						
Foot Length	25.88 ± 1.91	25.96 ± 1.85	0.99	0.99 – 1.00	0.11	0.32
Midfoot Width WB	8.79 ± 0.94	8.85 ± 0.89	0.99	0.97 – 1.00	0.09	0.25
Dorsal Arch Hgt WB	6.49 ± 0.54	6.48 ± 0.51	0.99	0.97 – 1.00	0.05	0.15
Midfoot Width NWB	7.71 ± 0.68	7.67 ± 0.70	0.98	0.95 – 0.99	0.09	0.26
Dorsal Arch Hgt NWB	8.08 ± 0.66	8.06 ± 0.65	0.99	0.97 – 1.00	0.07	0.19
Diff Midfoot Width	1.08 ± 0.34	1.18 ± 0.26	0.82	0.60 – 0.94	0.13	0.35
Diff Dorsal Arch Hgt	1.60 ± 0.18	1.59 ± 0.22	0.92	0.80 – 0.97	0.06	0.16
Foot Mobility Magnitude	1.94 ± 0.31	1.99 ± 0.28	0.91	0.79 – 0.97	0.09	0.21

**Note**: WB = Weight Bearing; NWB = Non-Weight Bearing; SD = Standard deviation, 95% CI = 95% Confidence Interval

**Table 5:** Descriptive statistics, standard error of the measurement (SEM), and minimal detectable change (MDC95%) scores for FEMALES (n = 211)

	**Mean ± SD** **(cm)**	**Range**	**SEM** **(cm)**	**MDC95%** **(cm)**
**LEFT FOOT**				
				
Foot Length	24.32 ± 1.24	21.20 – 28.30	0.08	0.22
Midfoot Width WB	7.92 ± 0.56	6.54 – 9.67	0.08	0.22
Dorsal Arch Hgt WB	6.18 ± 0.46	4.91 – 7.32	0.06	0.17
Midfoot Width NWB	6.99 ± 0.42	5.72 – 8.13	0.04	0.11
Dorsal Arch Hgt NWB	7.36 ± 0.49	5.81 – 8.82	0.06	0.17
Diff Midfoot Width	0.92 ± 0.32	0.08 – 2.10	0.11	0.32
Diff Dorsal Arch Hgt	1.19 ± 0.25	0.54 – 1.87	0.07	0.20
Foot Mobility Magnitude	1.52 ± 0.32	0.68 – 2.45	0.08	0.22
				
**RIGHT FOOT**				
				
Foot Length	24.32 ± 1.24	21.40 – 28.00	0.07	0.19
Midfoot Width WB	7.96 ± 0.58	6.55 – 9.87	0.06	0.16
Dorsal Arch Hgt WB	6.07 ± 0.45	4.82 – 7.15	0.05	0.13
Midfoot Width NWB	7.08 ± 0.44	5.67 – 8.31	0.06	0.17
Dorsal Arch Hgt NWB	7.35 ± 0.49	6.01 – 8.78	0.05	0.15
Diff Midfoot Width	0.88 ± 0.33	0.20 – 2.01	0.14	0.39
Diff Dorsal Arch Hgt	1.28 ± 0.25	0.68 – 1.94	0.07	0.20
Foot Mobility Magnitude	1.58 ± 0.31	0.91 – 2.37	0.09	0.25

**Note**: WB = Weight Bearing; NWB = Non-Weight Bearing; SD = Standard deviation

**Table 6:** Descriptive statistics, standard error of the measurement (SEM), and minimal detectable change (MDC95%) scores for MALES (n = 134)

	**Mean ± SD** **(cm)**	**Range**	**SEM** **(cm)**	**MDC95%** **(cm)**
**LEFT FOOT**				
				
Foot Length	26.87 ± 1.38	24.00 – 31.70	0.09	0.24
Midfoot Width WB	8.98 ± 0.65	7.58 – 11.05	0.09	0.26
Dorsal Arch Hgt WB	6.87 ± 0.47	5.73 – 7.93	0.06	0.18
Midfoot Width NWB	7.97 ± 0.45	6.93 – 9.27	0.04	0.12
Dorsal Arch Hgt NWB	8.12 ± 0.54	6.83 – 9.65	0.07	0.19
Diff Midfoot Width	1.02 ± 0.34	0.23 – 1.91	0.12	0.34
Diff Dorsal Arch Hgt	1.26 ± 0.25	0.62 – 1.94	0.07	0.21
Foot Mobility Magnitude	1.64 ± 0.30	0.94 – 2.60	0.08	0.22
				
**RIGHT FOOT**				
				
Foot Length	26.88 ± 1.39	23.80 – 31.70	0.08	0.21
Midfoot Width WB	9.03 ± 0.63	7.77 – 10.82	0.06	0.17
Dorsal Arch Hgt WB	6.77 ± 0.47	5.50 – 7.95	0.05	0.14
Midfoot Width NWB	8.03 ± 0.47	6.78 – 9.37	0.07	0.18
Dorsal Arch Hgt NWB	8.13 ± 0.55	6.89 – 9.67	0.06	0.17
Diff Midfoot Width	1.00 ± 0.32	0.13 – 1.79	0.14	0.38
Diff Dorsal Arch Hgt	1.35 ± 0.29	0.17 – 2.24	0.08	0.23
Foot Mobility Magnitude	1.70 ± 0.32	0.91 – 2.76	0.09	0.26

**Note**: WB = Weight Bearing; NWB = Non-Weight Bearing; SD = Standard deviation

The results of the Shapiro-Wilk goodness-of-fit test indicated all measurements for the female and male feet had a normal distribution. The results of the t tests between the left and right feet for females on all eight measurements indicated that only the DiffAH was significantly different (p < .0001). The DiffAH was also significantly different between the left and right feet for males (p < .006). Based on these findings, means, standard deviations, ranges and MDC95% for the normative values on the left and right feet for both females and males are listed in Table 5 and 6.

## Discussion

The present study was undertaken to evaluate a new composite measure of vertical and medial-lateral mobility of the midfoot called the foot mobility magnitude or FMM. The FMM is calculated using the change in dorsal arch height between weight bearing and non-weight bearing (DiffAH) as well as the change in midfoot width between weight bearing and non-weight bearing (DiffMFW). An advantage of these measurement techniques in comparison to previously described methods is that only a single mark is placed on the dorsum of the foot at the 50% point of total foot length. Thus, the need to palpate and mark bony landmarks that can lead to a reduction in reliability is avoided. Furthermore, the measure of the change in either the vertical or medial-lateral midfoot movement can be obtained without having to modify foot position through palpation (i.e., placement of foot in subtalar joint neutral position), which has also been shown to decrease the consistency of foot measurements. In addition to describing the measurement techniques used to calculate the FMM, another purpose of this study was to report on the intra-rater and inter-rater reliability of the measurements required to calculate the FMM. If the levels of intra-rater and inter-rater reliability were determined to be acceptable, then normative values for DiffAH, DiffMFW, and the Foot Mobility Magnitude (FMM) measurements would be provided.

The results demonstrate excellent levels of intra-rater and inter-rater reliability for all eight measurements assessed in the current study. The intra-rater ICC values for both within-session and between-session for all three raters ranged from 0.97 to 0.99. These ICC values would be classified as "almost perfect" for the within-session and between-session measurements made by all three raters. The difference in mean values between day 1 and day 2 for all three raters was less than 0.20 cm for all five measurements except midfoot width in weight bearing. The mean midfoot width in weight bearing values for rater 3 irrespective of the day measured, were 0.20 cm less than rater 1 and 0.36 cm less than rater 2. The mean difference in the mean midfoot width in weight bearing between rater 1 and rater 2 was 0.15 cm. The fact that rater 3 had the least amount of clinical experience in comparison to the other two raters, could indicate that clinical experience might be a factor in obtaining consistent measurements of DiffMFW. Just as important as the ICC results, the intra-rater SEM values were all less than 0.10 cm for both within-session and between-session for all three raters. Inter-rater reliability ICC values for the same five measurements, total foot length, midfoot width in weight bearing, dorsal arch height in weight bearing, midfoot width in non-weight bearing, dorsal arch height in non-weight bearing, also ranged from 0.97 to 0.99. The inter-rater reliability ICC values for the three calculated foot measures, DiffAH, DiffMFW, and FMM, ranged from 0.83 to 0.89. Based on these results, the inter-rater ICC values for all 8 measurements listed in Table 3 would also be classified as "almost perfect" using the characterizations provided by Landis and Koch [22]. As is noted in Table 3, the inter-rater SEM values for all 8 measurements were less that 0.15 cm. Based on these findings, the authors concluded that the reliability of the measurement techniques was acceptable and that further statistical analysis on the normative data could be performed.

The results of t tests indicated that of the eight measurements evaluated in this study, only the DiffAH was significantly different between the left and right feet for both the female and male subjects. The mean extremity difference in DiffAH, however, was less than 0.10 cm for both the female as well as male subjects. Although this small mean difference would not be considered a major dissimilarity from a clinical perspective, the authors of the current study decided against combining the left and right feet to create a larger data set since several issues have been raised regarding this practice. Menz has noted that the counting of the left and right feet as single independent observations artificially increases the data set by counting the same subject twice [27]. Furthermore, Menz states that it could be problematic to conduct research on individual feet rather than people since the manner in which an individual foot functions is at least partly dependent on the person to which the foot is attached [27]. In light of these issues, the remainder of the discussion of the results of the present study will focus on data specific to the 345 left and right feet.

In evaluating the normative data for the left and right feet of the 211 females and the 134 males, the SEM and MDC95% statistics provide the clinician and researcher with the ability to distinguish real changes in the measurements which are not likely attributed to chance variation in the measurement. Both statistics characterize the degree of measurement error in the same units as the measurements and are not affected by variability amongst individuals [28]. The SEM provides a direct representation of measurement error, while the MDC95% represents the minimum amount of change required to be 95% confident that the difference between a pre-and post- measurement is the result of real change [23]. The SEM values for the female left and right feet ranged from 0.04 to 0.14 cm with the MDC95% scores ranging from 0.11 to 0.39 cm. The SEM values for the male left and right feet also ranged from 0.04 to 0.14 cm with the MDC95% scores ranging from 0.12 to 0.38 cm. The DiffMFW measurement had the largest SEM and MDC95% values for both the females and the males.

In the current study, the mean values for DiffMFW for all 345 left and right feet were 0.96 and 0.93, respectively. While these values are slightly higher than the 0.70 cm mean for navicular drift on the 20 subjects reported by Vinicombe et al [16], they are quite similar to the 1.01 cm mean for navicular drift on the 26 subjects reported by Billis et al [17]. Although the DiffMFW measurement is not an actual measure of the medial-lateral movement of the navicular, the values obtained would appear to be similar to those values previously reported for navicular drift.

McPoil et al reported that the mean change in dorsal arch height between weight bearing and non-weight bearing was 1.00 cm [14]. In the current study, the mean values for DiffAH for all 345 left and right feet were 1.21 and 1.31 cm, respectively, which are larger than those previously reported by McPoil et al. It is important to note that different methodologies were used to measure dorsal arch height in the two studies. McPoil et al calculated both the weight bearing and non-weight bearing dorsal arch heights indirectly using digital images that were software enhanced and then printed so that measurements could be taken directly from the digital image. In the current study, the dorsal arch height for both weight bearing and non-weight bearing were measured directly on each foot, which not only permitted an immediate measurement but prevented the possibility of measurement error associated with distortion of the digital image.

In the present study, the MDC for the normative data was calculated using 95% confidence limits. Hopkins has suggested that 95% confidence limits are too stringent to use as a threshold for deciding that real change has occurred and instead recommends using 1.5 or 2.0 times the SEM rather than 2.77 times the SEM [25]. Thus, the use of 2.77 times the SEM in the current study could be considered a very conservative estimate of true change. For example, the MDC95% for the female left and right feet is 0.32 and 0.39 cm, respectively. Using 2 times the SEM as the measure of true change for the female left and right feet would be 0.24 and 0.28 cm, while 1.5 times the SEM would be 0.18 and 0.21 cm. While the ultimate decision on whether to use the more conservative MDC95% versus 1.5 or 2.0 times the SEM to determine the true change in measurement rests with the clinician or researcher, the normative data provided in this study allows the calculation of either criteria.

To the authors' knowledge, the FMM represents the first attempt to provide the clinician and researcher with a composite measure of both vertical and medial-lateral mobility of the midfoot. Although previous studies have described the need to assess mobility of the navicular bone in both the sagittal (vertical) and transverse (medial-lateral) planes, the measures of navicular drop and drift were specific and distinctive measurements. The advantage of the FMM is that it is based on two highly reproducible measurements of vertical (DiffAH) and medial-lateral (DiffMFW) mobility of the midfoot that can be collectively analyzed to create an overall index of midfoot mobility.

A limitation in the measurements proposed in this study to assess the change in the vertical and medial-lateral mobility of the midfoot is that they do not actually measure navicular drop and drift as has been previously defined in the literature. Based on radiographic data, the 50% of total foot length mark on the dorsum of the foot is positioned over the proximal 1/3 of the medial cuneiform. Wolf et al, however, have recently evaluated possible functional units of the foot to provide a basis for subdividing the entire foot for gait analysis. In their study, they utilized reflective marker arrays mounted on cortical pins that were inserted into seven bones of the foot in six healthy subjects [29]. The movement of the marker arrays was then analyzed for each subject during walking and slow running. Wolf et al reported that the navicular, medial cuneiform, and the first metatarsal performed as a functional unit with all three bones rotating in the same direction [29]. Based on their findings, even though the mark on the dorsum of the foot indicating 50% of total foot length is located on the proximal third of the medial cuneiform, it would appear that measuring the change in vertical and medial-lateral mobility of the midfoot at this location does provide an reasonable estimation of movement of the navicular as well as the medial cuneiform.

Another limitation of this study is that it was conducted entirely on asymptomatic individuals. As such, the normative values reported in this study may or may not be representative of individuals who have had an injury or who have some type of systemic disease such as rheumatoid arthritis. It does, however, provide normative values for each measurement that can be compared with those obtained in individuals with pathology.

## Conclusion

The findings of this study indicate that the measurements required to calculate the DiffAH, DiffMFW, and FMM are highly reproducible and provide the clinician with a method to quantify the vertical and medial-lateral mobility of the midfoot in comparison to the previously described measures of navicular drop and drift. The measurements described in this paper can be easily and efficiently performed in a clinical setting. In addition, normative data on a large group of healthy individuals is provided.

## Competing interests

The authors declare that they have no competing interests.

## Authors' contributions

TGM conceived the study, participated in the design of the study, carried out data collection and analysis. BV participated in the design of the study and carried out data collection. MWC participated in the design of the study and carried out analyses. NC carried out data collection. MW carried out data analyses.

## Consent

The Institutional Review Board of Northern Arizona University (IRB #06.0233) approved the protocol for data collection and all participants provided written informed consent prior to participation.
